# Medical management: a model for giving medical aid to children with infectious-inflammatory diseases of the urinary system

**DOI:** 10.25122/jml-2021-0104

**Published:** 2021

**Authors:** Volodymyr Volodymyrovych Bezruk, Igor Dmytrovych Shkrobanets, Oleksii Serhiiovych Godovanets, Oleksandr Hryhorovych Buriak, Nataliya Oleksandrivna Popelyuk, Nina Ivanivna Voytkevich, Olena Victorivna Makarova, Oksana Ivanivna Yurkiv, Michael Ivanovych Sheremet, Oleksandr Vyacheslavovich Boilookyi, Mykhailo Mykhailovich Hresko, Mariya Ivanivna Velia, Svyatoslava Vasylivna Yurniuk, Bogdana Petrivna Seniuk, Maryna Dmytrivna Hresko

**Affiliations:** 1. Department of Pediatrics, Neonatology and Perinatology Medicine, Bukovinian State Medical University, Chernivtsi, Ukraine; 2. Department of Medical and Organizational Management, National Academy of Medical Sciences of Ukraine, Kiev, Ukraine; 3. Department of Foreign Languages, Bukovinian State Medical University, Chernivtsi, Ukraine; 4. Department of Care for Patients and Higher Nursing Education, Bukovinian State Medical University, Chernivtsi, Ukraine; 5. Surgery Department No.1, Bukovinian State Medical University, Chernivtsi, Ukraine; 6. Department of Pharmacy, Bukovinian State Medical University, Chernivtsi, Ukraine; 7. Department of Internal Medicine, Bukovinian State Medical University, Chernivtsi, Ukraine; 8. Department of Obstetrics and Gynecology, Bukovinian State Medical University, Chernivtsi, Ukraine

**Keywords:** quality of the medical care, urinary tract infections, antibiotic resistance, children

## Abstract

Increasing requirements of medical aid given to children with infectious-inflammatory diseases of the urinary system stipulate the necessity to improve its quality using evidence-based therapeutic-diagnostic and organization technologies. The aim of the work – to substantiate, develop the improved model of the specialized nephrology care for children with infectious inflammatory diseases of the urinary system at the regional level. The official statistical data have been studied (2006 to 2017); information-analytical and statistical methods have been used. A bacteriological study (2009–2016) of urine samples was carried out for 3089 children (0–17 years old) in the Chernivtsi region. They formed the foundation for substantiation and development of an improved functional-organizational model of the system. In addition to the existing and functionally changed elements contains new elements: regional/inter-regional center of specialized medical aid to children with infectious-inflammatory diseases of the urinary system. Implementation of the elements of the suggested improved model in a part of a rational approach in distribution of functions concerning medical observation of patients at the stages of giving medical aid enabled to make the period of hospitalization of nephrological patients 11,40% shorter and an average period of treatment of patients with infectious-inflammatory diseases of the urinary system 2,93% shorter.

## Introduction

Infectious-inflammatory diseases of the urinary system organs are considered an urgent issue in pediatrics due to a high rate of occurrence among the children population and a considerable variation rate of the pathology in the structure of the total sickness of children from various regions of Ukraine [1–3].

Increasing requirements of medical aid given to children with infectious-inflammatory diseases of the urinary system stipulate the necessity to improve its quality using evidence-based therapeutic, diagnostic and organization technologies, an appropriate supply of resources including highly technological interventions in particular [4–7].

## Material and Methods

The aim of this study was to substantiate, develop the improved model of specialized nephrology care for children with infectious inflammatory diseases of the urinary system at the regional level. The official statistical data have been studied (reports on the state of medical care for children in the Chernivtsi region and data from the Center of Medical Statistics of the Ministry of Healthcare from 2006 to 2017), information-analytical and statistical methods have been used. The modern etiological structure of uropathogens – urinary tract infection (UTI) pathogens among the children of the Chernivtsi region (2009–2016) was studied. A bacteriological study of urine samples was carried out for 3089 children (0–17 years old) in the Chernivtsi region; the regional spectrum of sensitivity to antibacterial drugs was determined among the main groups of UTI pathogens; age, gender, and administrative-territorial differences among the children’s population of the region were analyzed. This work is a fragment of the scientific and research paper entitled “Scientific support, monitoring and evaluation of models of health care development in Ukraine at the regional level” (due date 2015–2017), state registration no. 0115U002852.

The approval for this study was obtained from the Ethics Committee of the HSEEU “Bukovinian State Medical University” and Chernivtsi Regional Endocrinology Center, Ukraine (approval ID: 15-07.12.2017).

## Results

Imperfect normative-legal regulation of the organization of medical aid given to children with urinary tract infection (UTI); lack of clear mechanisms of interaction between health care institutions including the primary, secondary and tertiary aid, out-patient and in-patient stages in giving specialized medical aid to children with infectious-inflammatory diseases of the urinary system in the region [8-11]; inadequate financing and imperfect existing organization structure of giving highly specialized highly technological medical aid to this group of population [12, 13]; untimely detection of diseases, ineffective use of specialized bed hospital stock, problems with availability of instrumental diagnostics, possibility to organize a qualitative observation over the patient depending on etiology of infection [14-23]; a low level of satisfaction among parents of children residing in the rural districts and those who were treated out-patiently in municipal health care institutions, support on the part of health care managers and doctors of the necessity to improve clinical routes of patients with infectious-inflammatory diseases of the urinary system at the regional level [24-25], found by the results of the study made the foundation for substantiation and development of an improved model of specialized medical aid given to children with infectious-inflammatory diseases of the urinary system at the regional level.

**Figure 1.**
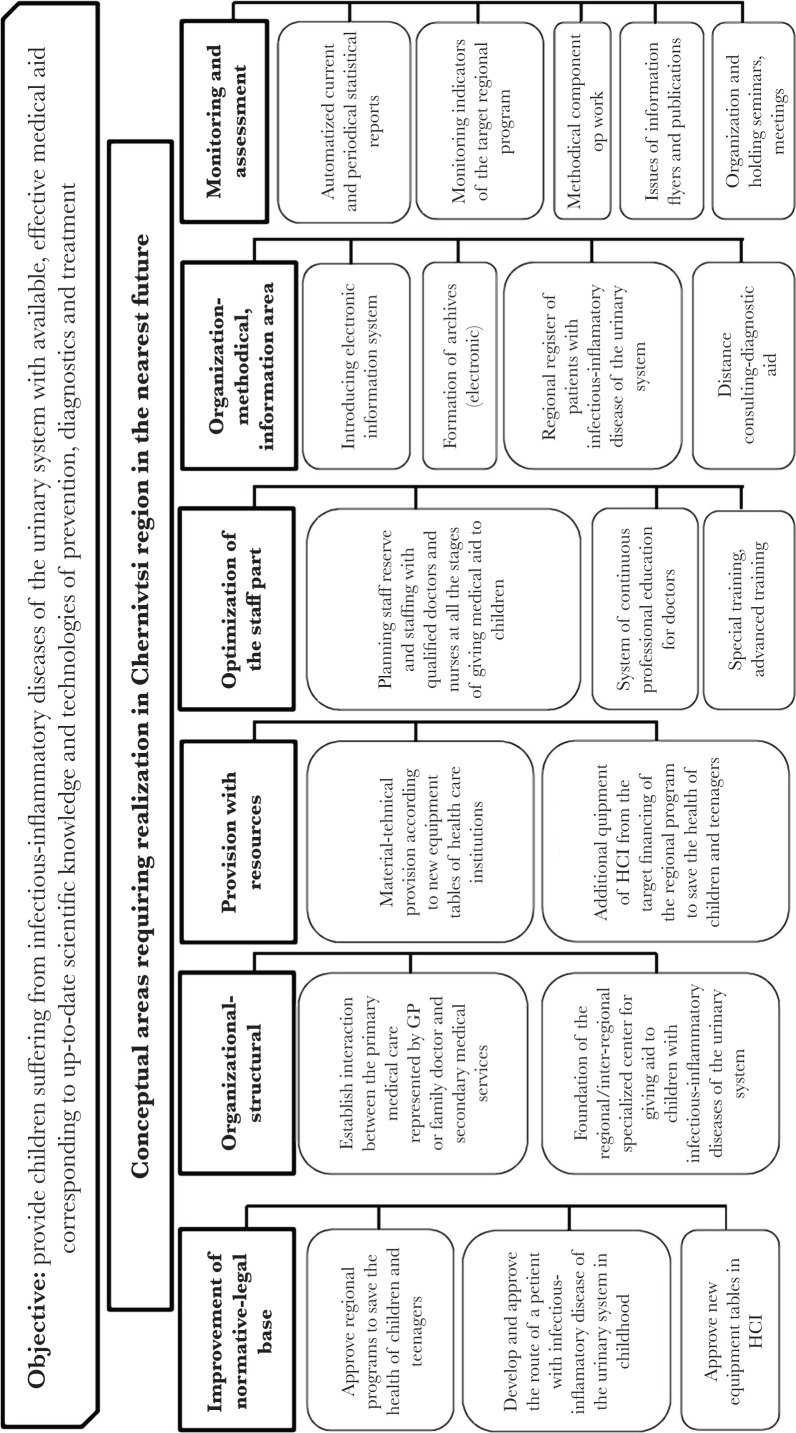
Conceptual areas to improve organization of giving medical aid to children with infectious-inflammatory diseases of the urinary system at the regional level (in terms of Chernivtsi region).

Conceptual areas to improve the standard of giving specialized medical aid to children with infectious-inflammatory diseases of the urinary system at the regional level have been substantiated by the results of the study (Figure 1).

**Figure 2.**
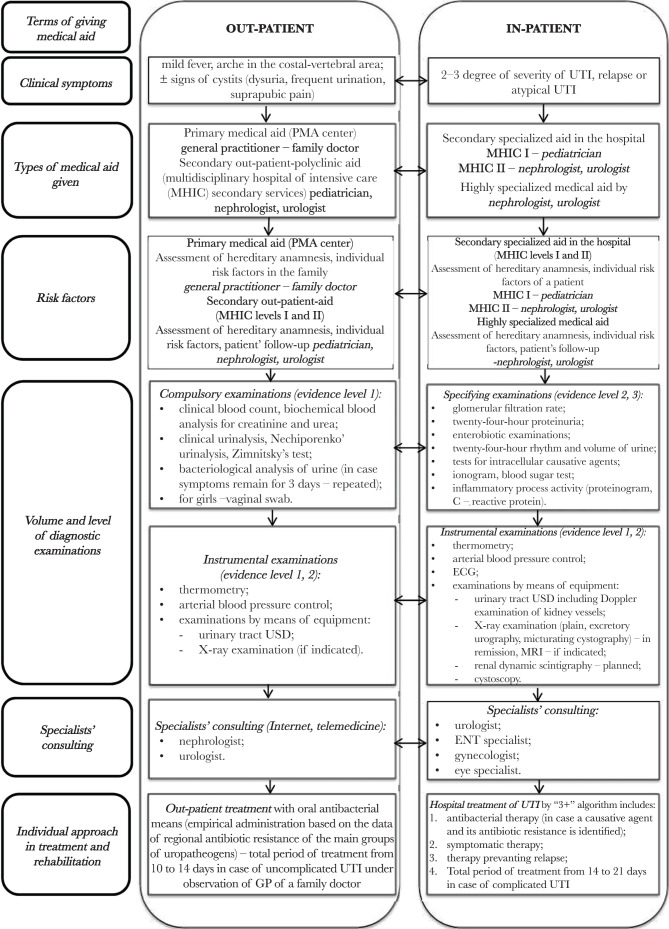
Medical-organizational technology to form the routes of patients with infectious-inflammatory diseases of the urinary system.

On the basis of the existing normative-legal documents regulating standardization of medical aid, clinical recommendations of the European Association of Urology (2012, 2015), and the data of our study, a clinical route of a patient aged from 0 to 17 years with infectious-inflammatory diseases of the urinary system is developed and implemented at the regional level into the work of health care institutions in Chernivtsi region which determine stages of giving medical aid (Figure 2).

A central element of the system and, in fact, an innovation element of the improved model is a regional/inter-regional center of specialized and highly specialized medical aid given to children with infectious-inflammatory diseases of the urinary system. Its medical staff provides the implementation of information, methodical, therapeutic, medical-organizational work with monitoring of indicators to complete the regional program and other functions through comprehensive therapeutic-preventive, social-economical, hygienic, medical-social, information-educational measures. Thus, they provide completely organizational work depending on the determined area of the region.

The center’s medical structure has several units to provide children with infectious-inflammatory diseases of the urinary system with available and effective evidence-based medical aid corresponding to the best world patterns, keeping to the stages of giving medical aid according to the improved clinical route. Functional distribution of the center is the following: out-patient consulting rooms of doctors (pediatric nephrologist, pediatric urologist; psychologist and social worker if necessary); in-patient nephrological department with the priority of day-time in-patient departments with intensive care units and dialysis; specialized microbiological laboratory; department of information-methodical supply and monitoring.

The following main tasks of the regional/inter-regional center of specialized medical aid given to children with infectious-inflammatory diseases of the urinary system are determined:

• achievement of final diagnosis, choosing the tactics of treatment, its correction, if necessary, rehabilitation and further out-patient care of children with UTI;
• organizational-methodical, clinical dynamic control over the results of the therapeutic and rehabilitation technologies used while giving medical aid to children with UTI in the region; hemodialysis of newly organized beds, referring to the Institute of Nephrology, National Academy of Medical Sciences of Ukraine, (a state institution) if highly specialized in-patient medical aid is necessary;
• to monitor changes of the regional spectrum peculiarities of bacterial causative agents in the urine of patients with UTI in various groups of children considering their gender and age; to organize the continuous treatment of children in case of relapses and exacerbations, to refer patients to be examined by medical-social experts if necessary, sanitary-resort treatment; to obtain epidemiological remarks by sickness rates of children suffering from UTI, their occurrence, and efficiency of medical aid they got (based on the data from the regional electronic register of patients initiated in the center);
• achievement of organizational-methodical, informative, communicative functions of the center in work with medical professionals in the region, public organizations, mass media concerning prevention and early finding of children with UTI.

**Figure 3.**
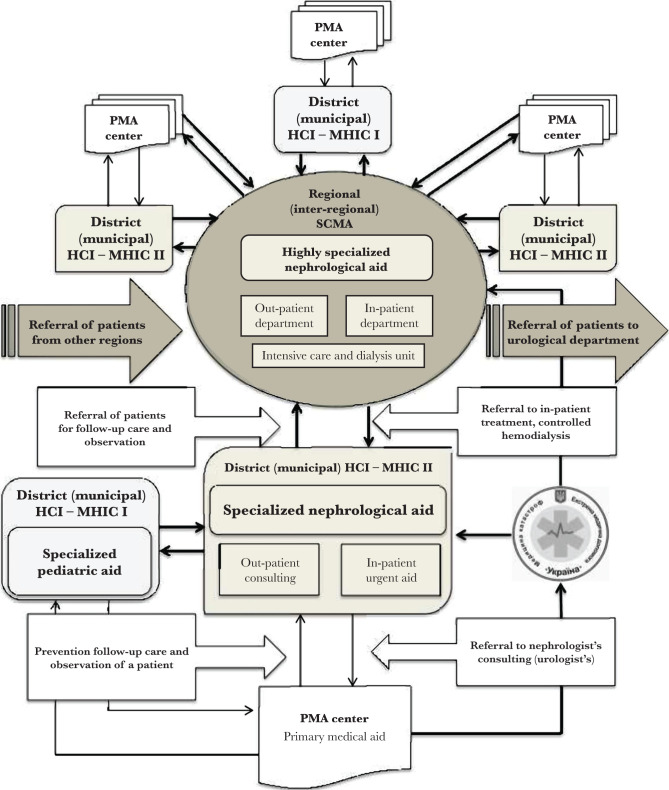
Stages to organize the interaction between the regional/inter-regional center and regional health care institutions giving medical aid to children.

The functions of the center include its organizational interaction with regional health care institutions giving medical aid to children and teenagers. The stages of this interaction are presented in Figure 3.

## Discussion

Therefore, the improved functional-organizational model of giving medical aid to children with infectious-inflammatory diseases of the urinary system at the regional level is based on a reformed medical aid system for the population. It consists of the following elements:

1. Already existing elements of the health care system giving medical aid to children with infectious-inflammatory urinary system diseases;
2. Existing but partially changed elements at the expense of their functional optimization such as pediatric service of the secondary health care institutions, microbiological laboratories of the health care institutions (HCI); interaction of HCI in giving various medical aid according to the improved clinical route; clinical and scientific base for the system of continuous postgraduate education of medical professionals giving medical aid to children with infectious-inflammatory diseases of the urinary system; first of all – general practitioners or family doctors, pediatricians, pediatric nephrologists and urologists;
3. The elements of new quality – the foundation of a regional/inter-regional center of specialized medical aid given to children with infectious-inflammatory diseases of the urinary system, introduction, and provision of work of the electronic register of patients, the electronic-information system as elements of the regional target comprehensive program for saving health of children in the Chernivtsi region. Results of the expert evaluation of the suggested innovations made by qualified independent experts were indicative of their importance in order to improve medical aid given to children with infectious-inflammatory diseases of the urinary system at the regional level by the major directions: systematic, comprehensive, stage-by-stage, successive approaches, and rational use of resources. On the whole, the suggested model was evaluated by the independent experts in the total sum of 9.26±0.08 points out of 10.0 possible, and coordinated agreement of the experts in making their decision (CV (%) = 2.50–5.07).

Therefore, under conditions of the current and prognosticated threats (reduced birth rate and high mortality rate of the population) [1, 26], disaggregated reforms of the health care system, lack of integrated medical space, and imbalance in the functioning of the branch, which do not provide adequate preconditions in order to obtain timely, effective, available and safe medical aid by the population [27–30], integration of the suggested elements with existing earlier and functionally changed ones, give new qualities to the improved pattern of giving medical aid to children with infectious-inflammatory diseases of the urinary system in order to ensure the ability of an appropriate medical aid required by the children in Ukraine.

## Conclusions

Implementation of the elements of the suggested improved model in a part of a rational approach in distribution of functions concerning the medical observation of patients at the stages of giving medical aid allowed making the period of hospitalization of nephrological patients and the average period of treatment of patients with infectious-inflammatory diseases of the urinary system shorter by 11.40% and 2.93%, respectively.

The efficiency of implementation of certain elements of the suggested model with its positive evaluation by independent experts and its compliance with the strategy of branch reforms enables to recommend the improved functional-organization model of giving medical aid to children with infectious-inflammatory diseases of the urinary system at the regional level to be introduced into the health care system of Ukraine.

## Acknowledgments

### Ethical approval

The approval for this study was obtained from the Ethics Committee of the Bukovinian State Medical University and Chernivtsi Regional Endocrinology Center, Ukraine (approval ID: 15-07.12.2017).

### Conflict of interest

The authors declare that there is no conflict of interest.
